# Prognostic Factors to Predict ICU Mortality in Patients with Severe ARDS Who Received Early and Prolonged Prone Positioning Therapy

**DOI:** 10.3390/jcm10112323

**Published:** 2021-05-26

**Authors:** Po-Hsin Lee, Chen-Tsung Kuo, Chiann-Yi Hsu, Shih-Pin Lin, Pin-Kuei Fu

**Affiliations:** 1 Division of Chest, Department of Internal Medicine, Taichung Veterans General Hospital, Taichung 407219, Taiwan; berry7bo@gmail.com; 2 Computer & Communications Center, Taipei Veterans General Hospital, Taipei 11217, Taiwan; jmskuo@gmail.com; 3 Biostatistics Task Force of Taichung Veterans General Hospital, Taichung 407219, Taiwan; chiann@vghtc.gov.tw; 4 Department of Critical Care Medicine, Taichung Veterans General Hospital, Taichung 407219, Taiwan; yalebin.lin@gmail.com; 5 Ph.D. Program in Translational Medicine, National Chung Hsing University, Taichung 402010, Taiwan; 6 College of Human Science and Social Innovation, Hungkuang University, Taichung 433304, Taiwan; 7 Department of Computer Science, Tunghai University, Taichung 407224, Taiwan

**Keywords:** acute respiratory distress syndrome, prone positioning, prognostic factors, ICU mortality

## Abstract

Early and prolonged prone positioning (PP) therapy improve survival in advanced ARDS; however, the predictors of mortality remain unclear. The study aims to identify predictive factors correlated with mortality and build-up the prognostic score in patients with severe ARDS who received early and prolonged PP therapy. A total of 116 patients were enrolled in this retrospective cohort study. Univariate and multivariate regression models were used to estimate the odds ratio (OR) of mortality. Factors associated with mortality were assessed by Cox regression analysis and presented as the hazard ratio (HR) and 95% CI. In the multivariate regression model, renal replacement therapy (RRT; OR: 4.05, 1.54–10.67), malignant comorbidity (OR: 8.86, 2.22–35.41), and non-influenza-related ARDS (OR: 5.17, 1.16–23.16) were significantly associated with ICU mortality. Age, RRT, non-influenza-related ARDS, malignant comorbidity, and APACHE II score were included in a composite prone score, which demonstrated an area under the curve of 0.816 for predicting mortality risk. In multivariable Cox proportional hazard model, prone score more than 3 points was significantly associated with ICU mortality (HR: 2.13, 1.12–4.07, *p* = 0.021). We suggest prone score ≥3 points could be a good predictor for mortality in severe ARDS received PP therapy.

## 1. Introduction

The prevalence of acute respiratory distress syndrome (ARDS) in patients admitted to the intensive care units (ICU) is approximately 10% [1], and the mortality rate ranges from 30% to 50% because of the high heterogeneity in ARDS [2,3]. Although pharmaceutical treatment is limited, prone positioning (PP) improves the outcomes of patients with ARDS [3,4,5]. In 1976, Piehl and Brown proposed that PP therapy could improve oxygenation in patients with ARDS [6]. Since 2001, several randomized control trials have demonstrated the survival benefit of PP therapy in patients with ARDS [7,8,9,10,11,12,13,14,15,16,17,18]. In 2013, The PROSEVA trial demonstrated that early application and prolonged duration of PP significantly reduced mortality in patients with moderate to severe ARDS [7]. Since then, five meta-analyses have recommended that inpatients with ARDS requiring PP therapy, early introduction of PP accompanied by lung protective strategy and prolonged PP to ≥10–12 h per day were associated with lower mortality [19,20,21,22,23].

PP has become a standard treatment in ARDS, and numerous studies have revealed factors associated with lower mortality [4,5,24]. However, few studies have discussed the poor prognostic factors in patients with ARDS who received early and prolonged PP therapy [24,25]. Modrykamien et al. analyzed 43 patients with severe ARDS treated with PP and found that only three parameters were significant predictors of survival in ICU: APACHE II score, plateau pressure (Pplat), and driving pressure [24]. Kao et al. enrolled 65 patients with severe influenza-related pulmonary ARDS and found three factors to be independently associated with 60-day mortality: pneumonia severe index, renal replacement therapy (RRT), and dynamic change in driving pressure [25]. However, these prognostic factors are of limited clinical utility in predicting which patients will benefit from PP therapy because lack of standardized PP protocols, only on influenza-related ARDS, lack of consideration of ICU mortality, and lack of a scoring system for clinical application [24,25]. In the current study, we identified factors associated ICU mortality in 116 patients with severe ARDS who received early and prolonged PP therapy and developed a prognostic score (prone score) to predict ICU mortality [20].

## 2. Materials and Methods

### 2.1. Study Design and Patients

This retrospective cohort study was conducted in the medical ICUs of Taichung Veterans General Hospital (TCVGH), a 1200-bed tertiary referral center in Taiwan, from January 2015 to June 2018. We enrolled patients diagnosed as having ARDS who received mechanical ventilation in ICUs and were treated with PP for moderate to severe hypoxemia despite a positive end-expiratory pressure (PEEP) of >10 cmH2O. Severe hypoxemia was defined as a PaO2/fraction concentration of inspired oxygen (FiO2) ratio (PF ratio) < 100 mmHg according to previous clinical trials [26,27] and the Berlin definition of ARDS [27]. We excluded patients who received PP therapy for <6 h and those who received extracorporeal membrane oxygenation (ECMO) within 48 h due to failed PP therapy or comorbid with poor cardiac function. Data related to demographics, laboratory examination, period from hypoxemia to PP, duration of PP therapy, ventilator settings, comorbidities, and clinical outcomes were extracted from the electronic medical records. The need for patient consent was waived due to the retrospective study design and anonymization and deidentification of patient data prior to analysis. All methods were performed in accordance with the declaration of Helsinki, relevant guidelines and regulations. The study was reviewed and waived the patient consent was approved by the Institutional Review Board (IRB number, CE20308B; date of approval, 16 September 2020, Institutional Review Board-II, 109-B-11 Board Meeting) of Taichung Veterans General Hospital.

### 2.2. Mechanical Ventilator Setting, Recruitment Maneuver, and Protocol of PP Therapy

Patients diagnosed ARDS were treated with lung protective strategy to maintain Pplat ≤ 30 cmH2O by using lower tidal volume ventilation (goal of tidal volume: 4–6 mL/kg predicted body weight). The setting of PEEP in our ICUs was followed by a lower PEEP strategy according to previous research and meta-analysis [28,29,30]. FiO2 in the ventilator was adjusted to keep oxyhemoglobin saturation by pulse oximetry (SpO2) > 90%. The PEEP-FiO2 combinations were the following: 5–8/0.5, 8–10/0.6, 10–12/0.7, 12–14/0.8, 14–16/0.9, and 16–18/1.0 [31]. For patients who failed to maintain the goal of SpO2 ≥ 90% even using FiO2 of >0.6, recruitment maneuvers (RMs) were indicated through brief application of a high level of continuous positive airway pressure (CPAP) to correct hypoxemia. In our hospital, RMs include sustained inflation by abruptly raising the CPAP to 40 cmH2O for 40 s [32,33]. PP was initiated as rescue therapy for patients with ARDS who experienced refractory hypoxemia within 24 h, provided the following criteria were met: PaO2/FiO2 < 150 mmHg, FiO2 ≥ 0.6, and PEEP ≥ 10 cmH2O.

The protocol of PP therapy was according to previous publication [31]. In brief, patients lied in a prone position on a silicone pad, with their dependent parts supported by silicone cushions. Patients received PP continuously for 48–72 h and even longer once PaO2/FiO2 remained <150. During PP therapy, patients were turned right or left alternately every 2 h to avoid pressure sore formation. After hypoxemia improved and clinical condition stabilized (i.e., when SpO2 > 90% and FiO2 < 60% for >24 h after at least 48–72 h of PP therapy), patients lied in the supine position.

### 2.3. Data Collection, Assessment, and Outcome Measures

Data were collected on age, sex, body mass index (BMI), Acute Physiology and Chronic Health Evaluation II (APACHE II) score, and major comorbidities. The major comorbidities were identified using the International Classification of Diseases, Ninth Revision, Clinical Modification (ICD-9-CM) code, such as congestive heart failure (CHF), coronary artery disease (CAD), interstitial lung disease (ILD), chronic obstructive pulmonary disease (COPD), diabetes mellitus (DM), chronic kidney disease (CKD), liver cirrhosis, autoimmune disease, and malignancy. Patients who received RRT during PP therapy were identified and analyzed. Parameters of ventilator settings extracted from electronic medical records included the following: mode of ventilation, tidal volume, peak inspiratory pressure (PIP), PEEP, and Pplat. The primary outcome was ICU mortality, which was defined as death in the ICU.

### 2.4. Statistical Analysis

Statistical analyses and database management were performed using SPSS (version 22.0; IBM, Armonk, NY, USA). Categorical variables are presented as frequencies and percentages and were analyzed with the chi-square test. Nonparametric data were assessed using the Mann–Whitney U test and are presented as the median and interquartile range (IQR). Univariate and multivariate logistic regression models were used to estimate the odds ratio (OR). Receiver operating characteristic (ROC) curve analysis were performed for all the parameters measured and the cut-off points were decided to maximize the sum of sensitivity and specificity values of the respective ROC curves. Factors associated with mortality was assessed by Cox proportional hazard model. The strength of association is presented as the Hazard ratio (HR) and 95% CI. In this study, we used the two-tailed test, and significance was set at *p* < 0.05.

## 3. Results

**Figure 1:**
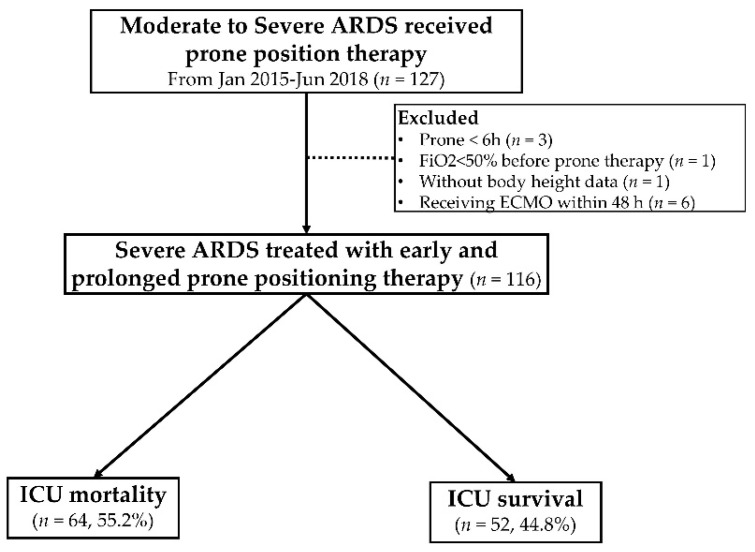
Enrollment and follow-up of the study participants. ARDS, acute respiratory distress syndrome; FiO2, fraction concentration of inspired oxygen; ECMO, extracorporeal membrane oxygenation; ICU: intensive care unit.

From January 2015 to June 2018, 116 patients with ARDS received mechanical ventilation in ICUs and were treated with PP for severe hypoxemia despite using PEEP of >10 cmH2O (Figure 1). Table 1 presents the patients’ demographic characteristics, etiology of ARDS, comorbidities, protocol of PP therapy, ventilator parameters, and ICU mortality (Table 1). The time from diagnosis of ARDS to initiation of PP therapy was 18.2 (IQR, 8.2–34.0) h, and the duration of continuous PP therapy was 66.1 (IQR, 44.2–85.3) h, and the median PF ratio was 90.8 (IQR, 70.5–114.0) which fit the current treatment concept of early and prolonged PP in severe ARDS (Table 1). In this cohort, the major cause of ARDS was noninfluenza-related ARDS (*n* = 83, 71.6%), followed by influenza-related ARDS (*n* = 20, 17.2%) and extrapulmonary ARDS (*n* = 13, 11.2%). The median APACHE II score was 31, indicating high clinical severity in this cohort. The ICU mortality was 55.2% (*n* = 64). Figure 1 presents the details of enrollment and follow-up.

Clinicodemographic parameters were compared between surviving and nonsurviving patients (Table 2). The nonsurviving patients were older, had a malignant comorbidity, had a higher APACHE II score, noninfluenza-related pulmonary ARDS and received RRT more frequently (all *p* < 0.05). Other variables were not significantly different between the surviving and nonsurviving groups (Table 2).

Table 3 summarizes the results of logistic regression analysis for determining factors associated with ICU mortality. Univariate analysis identified five factors associated with mortality: age (OR, 1.04; 95% CI, 1.01–1.07, *p* = 0.003), APACHE II score (OR, 1.07; 95% CI, 1.01–1.14; *p* = 0.029), noninfluenza-related pulmonary ARDS (OR, 3.78 compared with extrapulmonary ARDS; 95% CI, 1.07–13.29; *p* = 0.039), RRT (OR, 3.38; 95% CI, 1.55–7.36; *p* = 0.002), and active malignant disease (OR, 7.42; 95% CI, 2.06–26.7; *p* = 0.002). Multivariate analysis indicated that noninfluenza-related pulmonary ARDS (OR: 5.17 compared with extrapulmonary ARDS, 95% CI: 1.16–23.16), RRT (OR, 4.05; 95% CI, 1.54–10.67; *p* = 0.005), and active malignant disease (OR, 8.86; 95% CI, 2.22–35.41; *p* = 0.003) were significant predictive factors of ARDS-related mortality (Table 3).

**Figure 2:**
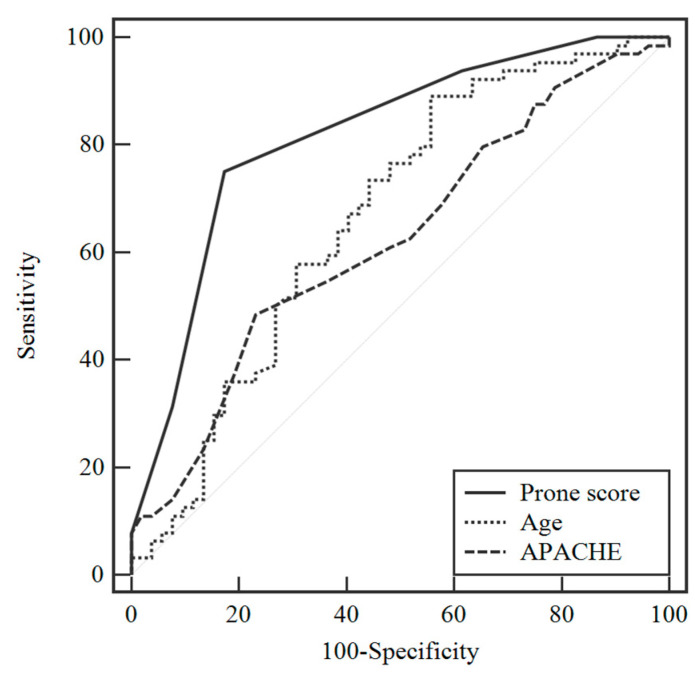
Receiver operating curves analysis of age, APACHE II score, and prone score for predicting ICU mortality in patients with acute respiratory distress syndrome receiving early and prolonged prone positioning.

**Figure 3:**
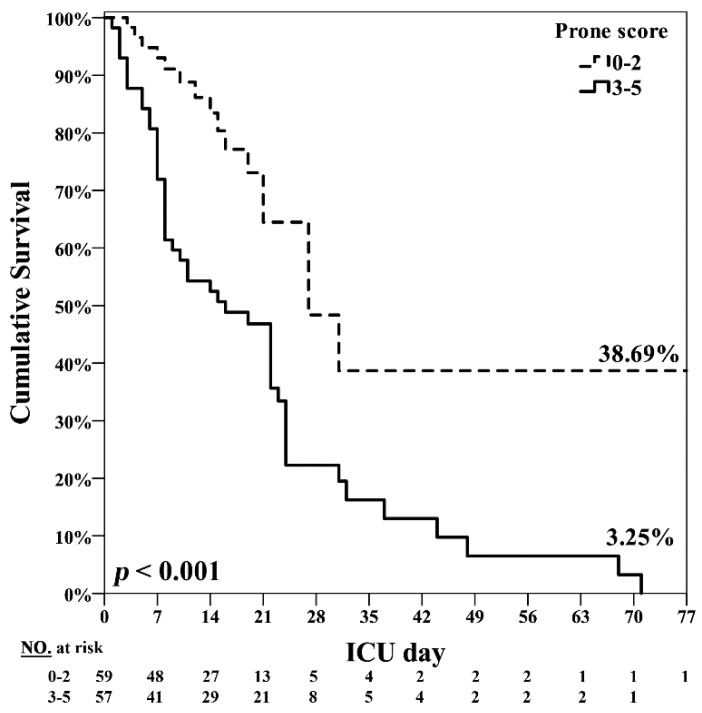
Prone score ≥ 3 was significantly associated with higher mortality in patients with severe ARDS received prone positioning therapy. *p* < 0.001.

A “prone score” was generated to predict the risk of ICU mortality. Receiver operating curves (ROC) were plotted to identify the optimal cutoff threshold in each parameter for predicting ICU mortality (Figure 2). The cutoffs were the following: age, 53 years (AUC = 0.668) and APACHE II score, 33 points (AUC = 0.623) (Figure 2 and Supplementary Table S1). The composite “prone score” to predict poor prognosis included five parameters: (1) age ≥ 53 years, (2) APACHE II ≥ 33, (3) receiving RRT in the ICU, (4) noninfluenza-related pulmonary ARDS, and (5) malignancy. Each item was assigned 1 point, and the total prone score ranged from 0 to 5 points. The cutoff value of the prone score with the best predictive power of ICU mortality was ≥ 3 points (AUC = 0.816), which was better than the APACHE II score (Supplementary Table S1). The sensitivity of prone score to predict ICU mortality was 75.0% and the specificity was 82.7% (Supplementary Table S1). Univariate analysis by logistic regression analysis revealed that prone score ≥ 3 points had a significantly higher risk of ICU mortality (OR, 14.33; 95% CI, 5.74–35.77; *p* < 0.001; Table 3). By using Cox regression model to analyze factors associated with ICU mortality, we found that age (HR, 1.02; 95% CI, 1.00–1.03), APACHE II score (HR, 1.08; 95% CI, 1.03–1.13), malignancy (HR, 2.06; 95% CI, 1.20–3.52) and prone sore more than 3 points (HR, 2.84; 95% CI, 1.61–5.01) were associated ICU mortality (Table 4). By multivariate analysis, prone score was the independent factors associated with ICU mortality (HR, 2.13; 95% CI, 1.12–4.07). In this cohort, we found that prone score ≥ 3 was significantly associated with higher mortality in patients with severe ARDS received prone positioning therapy. *p* < 0.001 (Figure 3).

## 4. Discussion

The current study had three major findings. First, we identified five factors associated with ICU mortality in patients with severe ARDS who received PP therapy: age, higher APACHEII score, noninfluenza-related pulmonary ARDS, RRT, and malignant comorbidity. Second, we developed a new clinical scoring tool—the prone score—to predict refractoriness to PP therapy and high risk of ICU mortality due to advanced ARDS. Third, our data revealed that the risk of ICU mortality in patients with higher prone score (3–5 points) was 2.13 times higher than those with lower prone score (1–2 points). This is the first real-world study to evaluate the treatment outcomes of PP therapy in patients with severe ARDS and develop a prediction score of ICU mortality in them.

Factors associated with good outcomes for these patients are early PP initiation, prolonged PP treatment sessions, and combinations with lung protective strategies [7,13]. However, how early should PP therapy be initiated to reduce mortality in ARDS remains unclear. Guérin et al. (2004) proposed that PP therapy should be initiated “as early as possible” in patients with ARDS [7], which has been widely followed, with PP being initiated between 6 and 72 h after ARDS diagnosis [3,7,9,12,13,17,18]. In 2013, the PROSEVA trial clarified that PP therapy should be initiated within 36 h of ARDS diagnosis [3]. In our medical ICUs, the protocol is PP initiation within 24 h of the diagnosis of moderate to severe ARDS. In the current study, the timing of PP initiation was within 36 h (median: 18.3 h, IQR: 8.4–33.4). In addition, no significant difference between the surviving and nonsurviving groups regarding the timing of PP initiation was noted (15.6 vs. 21.3 h, *p* = 0.084). Since our policy was arranged early and prolonged PP for all the patients with moderate to severe ARDS, therefore, the timing of PP therapy is less likely to be a confounder in ICU mortality in this cohort.

Prolonged PP therapy maybe a critical factor associated with mortality [7,9,17,18]. In the PROSEVA trial, the goal of PP therapy was >20-h duration, and the actual dose was 17 h on average, which reduced mortality in patients with moderate to severe ARDS [7]. Two meta-analyses stated that the PP therapy >12 h/day significantly reduced mortality in patients with ARDS having refractory hypoxemia [19,23]. In our medical ICUs, patients received PP continuously for 48–72 h and even longer if PaO2/FiO2 persisted to be <150 [31]. A recent prospective study performed 231 PP sessions with a mean length of 21.5 ± 5 h per session in patients with ARDS and recommended that the duration of PP therapy should be ≥24 h, depending on whether PaO2/FiO2 remains <150 [34]. In the current study, PP therapy was significantly longer than 20 h (median: 66.1 h, IQR: 44.4–85.4), with no significant difference between the surviving and nonsurviving groups (69.3 vs. 61.1 h, *p* = 0.170). Therefore, the dose of PP therapy is less likely to be a confounder in ICU mortality in this cohort.

Lung protective strategy in conjunction with PP appeared to be a useful approach. Two meta-analyses revealed that the benefit of PP therapy in reducing mortality was only found in combination with the lung protective strategy [19,20]. At our ICUs, the lung protective strategy is the standard of care. Therefore, in the current study, ventilation settings, including tidal volume, PEEP, and Pplat, were not significantly different between the surviving and nonsurviving groups.

The main strength of this study is the development of a scoring system to evaluate patients with severe ARDS admitted to the ICUs to determine who will receive greater benefit from early and prolonged PP therapy. Several studies have attempted to determine factors associated with mortality in patients with ARDS, including those receiving PP therapy. Lim et al. found that oxygenation improved faster in patients with extrapulmonary ARDS than pneumonia-related ARDS [35]. Kao et al. retrospectively examined 65 patients with influenza-related ARDS treated with PP and found that 60-day mortality was associated with a higher pneumonia severity index, RRT, and increased dynamic driving pressure [25]. Modrykamien et al. retrospectively studied 43 patients and identified only three factors—APACHE II score, Pplat, and driving pressure—to be associated with mortality when receiving PP therapy [24]. These studies provided some clues of mortality predictors but were limited in developing a scoring system. In our study, we identified five prognostic factors from 116 patients and used their cutoffs to develop the prone score to predict ICU mortality: age ≥ 53 years, APACHE II score ≥ 33 points, receiving RRT, malignant comorbidity, and noninfluenza-related ARDS.

This study has several limitations. First, because of the retrospective design, heterogeneity may have existed in each patient. Second, the study was conducted at medical ICUs in a single medical center rather than a multicenter study, meaning that the results may not be generalizable. In practice, the protocol of PP therapy, especially timing of initiation and dosage, and combination with other intensive respiratory therapies, such as recruitment maneuver and fluid strategy, vary between ICUs in different hospitals. In our medical ICUs, we followed a standard protocol of early and prolonged PP therapy since 2007 [31]. We treated patients with sepsis, pneumonia, and ARDS by using the documented protocol modified from the latest severe sepsis guideline [36]. In addition, our ICUs were serviced by full-time intensivists. Therefore, heterogeneity in ICU care and PP therapy protocols was minimal in the present study. Third, we excluded six patients who received PP therapy for <6 h and those who received extracorporeal membrane oxygenation (ECMO) within 48 h due to failed PP therapy or comorbid with poor cardiac function. Therefore, the study may exist a selection bias. Forth, critically ill patients in different countries or ICUs may have different demographic patterns, disease severity, and comorbidities, which can confound ICU mortality. Finally, our results may not be generalizable to patients with ARDS in pediatric, neurosurgical, surgical, and cardiac ICUs, because the current study included only adult patients admitted to the adult medical ICUs in TCVGH. Validation of prone score by a multicenter study is warrant in the future.

## 5. Conclusions

We developed the prone score to predict ICU mortality in patients with ARDS receiving early and prolonged PP therapy. The prone score comprises five parameters: age ≥ 53 years, receiving RRT in ICU, noninfluenza-related pulmonary ARDS, malignancy, and APACHE II score ≥ 33. The corresponding mortality rates for low risk (score 0–2) and high risk (score 3–5) were 27.1% and 84.2%, respectively. Prone score more than 3 points was the independent factor associated ICU mortality (HR: 2.13, 1.12–4.07, *p* = 0.021). To the best of our knowledge, the current study is the first article to develop the prognostic score for patients with ARDS receiving early and prolonged PP therapy.

## Figures and Tables

**Table 1:** Demographic characteristics, severity scores, comorbidities and clinical outcomes of patients with severe ARDS receiving early and prolonged prone positioning therapy in the intensive care unit (*n* = 116).

Variables	Median	IQR
Age (year)	62.9	51.8–74.5
Gender-Male (*n*, %)	70	60.3%
Body mass index (kg/m^2^)	24.4	21.9–27.9
APACHE II score	31.0	27.0–35.0
Cause of ARDS		
Non-influenza pulmonary ARDS (*n*, %)	83	71.6%
Influenza (*n*, %)	20	17.2%
Extrapulmonary ARDS (*n*, %)	13	11.2%
Renal replacement therapy (*n*, %)	52	44.8
Comorbidities (*n*, %)		
Congestive heart failure	6	5.2%
Coronary artery disease	8	6.9%
Interstitial lung disease	10	5.2%
Chronic obstructive lung disease	40	34.5%
Diabetes mellitus	44	37.9%
Chronic kidney disease	44	37.9%
Liver cirrhosis	10	8.6%
Autoimmune disease	18	15.5%
Malignancy	23	19.8%
Prone information		
Timing from ARDS to prone (h)	18.2	8.2–34.0
Total prone duration (h)	66.1	44.2–85.3
PaO2/FiO2 (PF ratio)	90.8	70.5–114.0
Ventilator setting		
Tidal volume (mL/kg)	6.0	5.7–6.5
PEEP (cmH_2_O)	14.0	14–16
PIP (cmH_2_O)	32.0	29–35
Pplat (cmH_2_O)	29.0	26.2–31
Driving pressure (cmH_2_O)	13.0	11.8–16.2
Compliance (mL/cmH_2_O)	25.8	21.2–32.8
ICU mortality (*n*, %)	64	55.2%

Continuous data are expressed as median and IQR. Categorical data were expressed number and percentage. CHF, congestive heart failure; CAD, coronary artery disease; ILD, interstitial lung disease; COPD, chronic obstructive pulmonary disease, DM, diabetes mellitus; CKD, chronic kidney disease; PEEP: positive end-expiratory pressure; PIP, peak inspiratory pressure; Pplat, plateau pressure.

**Table 2:** Parameters of patients with ARDS receiving prone positioning stratified by survival status.

Characteristics	Alive (*n* = 52; 44.8%)	Death (*n* = 64; 55.2%)	*p* Value
Age (years)	56.7 (46.1–68.3)	65.7(57.2–76.3)	0.002 **
Sex-Male, *n* (%)	34 (65.4)	36 (56.3)	0.418
BMI (kg/m^2^)	25.5 (22.0–28.4)	24.0 (21.7–27.1)	0.289
APACHE II score	30.0 (25.3–32.0)	32.0 (28.0–35.0)	0.022 *
Cause of ARDS			0.032 *
Non-influenza pulmonary ARDS	31 (59.6%)	52 (81.3%)	
Influenza	12 (23.1%)	8 (12.5%)	
Extra-pulmonary ARDS	9 (17.3%)	4 (6.3%)	
Renal replacement therapy	15 (28.8%)	37 (57.8%)	0.003 **
Comorbidity			
Congestive heart failure ^f^	4 (7.7%)	2 (3.1%)	0.406
Coronary artery disease ^f^	4 (7.7%)	4 (6.3%)	1.000
Interstitial lung disease ^f^	3 (5.8%)	3 (4.7%)	1.000
Chronic obstructive lung disease ^f^	6 (11.5%)	4 (6.3%)	0.340
Diabetes mellitus	22 (42.3%)	18 (28.1%)	0.161
Chronic kidney disease	17 (32.7%)	27 (42.2%)	0.392
Liver cirrhosis ^f^	3 (5.8%)	7 (10.9%)	0.508
Autoimmune disease	7 (13.5%)	11 (17.2%)	0.769
Malignancy	3 (5.8%)	20 (31.3%)	0.00 **
Prone information			
Timing from ARDS to prone (h)	15.6 (7.5–30.6)	21.3 (9.0–47.8)	0.084
Total prone duration (h)	69.3 (51.7–85.4)	66.1 (34.4–85.3)	0.170
PaO2/FiO2 (PF ratio)	93.4 (69.1–119.6)	88.7 (72.0–112.6)	0.368
Ventilator setting			
Tidal volume (mL/kg)	5.9 (5.7–6.3)	6.2 (5.8–6.6)	0.088
PEEP (cmH_2_O)	16.0 (14.0–16.0)	14.0 (14.0–16.0)	0.108
PIP (cmH_2_O)	32.0 (29.1–35.0)	32.5 (29.0–35.0)	0.953
Pplat (cmH_2_O)	28.0 (26.4–30.8)	29.0 (26.0–31.0)	0.765
Driving pressure (cmH_2_O)	13.0 (11.0–16.5)	14.0 (12.0–16.6)	0.349
Compliance (mL/cmH_2_O)	26.4 (22.6–33.8)	25.4 (20.0–30.8)	0.252

Mann-Whitney U test. Chi-Square test. ^f^ Fisher’s exact test. * *p* < 0.05, ** *p* < 0.01. Continuous data are expressed as median and IQR. Categorical data are expressed number and percentage. APACHE II: Acute Physiology and Chronic Health Evaluation II; CHF, congestive heart failure; CAD, coronary artery disease; CKD, chronic kidney disease; COPD, chronic obstructive pulmonary disease, DM, diabetes mellitus; ILD, interstitial lung disease; PEEP: positive end-expiratory pressure; PIP, peak inspiratory pressure; Pplat, plateau pressure.

**Table 3:** Logistic regression analysis of clinical variables associated with ICU mortality in patients with ARDS receiving early and prolonged prone positioning.

Variables	Univariate Analysis OR (95% CI)	Multivariate Analysis OR (95% CI)
Age (year)	1.04 (1.01–1.07) **	1.02 (0.99–1.05)
APACHE II score	1.07 (1.01–1.14) **	1.05 (0.97–1.14)
Cause of ARDS		
Extrapulmonary ARDS	Reference	Reference
Non-influenza pulmonary ARDS	3.78 (1.07–13.29) *	5.17 (1.16–23.16) *
Influenza ARDS	1.50 (0.34–6.58)	2.00 (0.36–11.12)
Renal replacement therapy	3.38 (1.55–7.36) **	8.86 (2.22–35.41) **
Comorbid with malignancy	7.42 (2.06–26.70) **	
Time from ARDS to prone (h)	1.01 (1.00–1.03)	
Total prone duration (h)	1.01 (0.99–1.01)	
PaO2/FiO2 (PF ratio)	0.99 (0.98–1.00)	
Prone score ≥ 3	14.33 (5.74–35.77) **	

Logistic regression. * *p* < 0.05, ** *p* < 0.01. OR, odds ratio; CI, confidence interval.

**Table 4:** Cox analysis of clinical variables associated with ICU mortality in patients with ARDS receiving early and prolonged prone positioning.

Variables	Univariate Analysis HR (95% CI)	Multivariate Analysis HR (95% CI)
Age (year)	1.02 (1.01–1.03) *	1.01 (0.99–1.03)
APACHE II score	1.08 (1.03–1.13) **	1.06 (1.01–1.11) *
Cause of ARDS		
Extrapulmonary ARDS	Reference	
Non-influenza pulmonary ARDS	1.07 (0.38–2.98)	
Influenza ARDS	0.93 (0.28–3.10)	
Renal replacement therapy	1.52 (0.92–2.51)	
Comorbid with malignancy	2.06 (1.20–3.52) **	
Time from ARDS to prone (h)	1.00 (1.00–1.01)	
Total prone duration (h)	1.00 (0.99–1.00)	
PaO2/FiO2 (PF ratio)	0.99 (0.99–1.00)	
Prone score ≥ 3	2.84 (1.61–5.01) **	2.13 (1.12–4.07) *

Cox regression. * *p* < 0.05, ** *p* < 0.01. HR, hazard ratio; CI, confidence interval.

## Data Availability

The data presented in this study are available on request from the corresponding author. The data are not publicly available due to the regulation of Institutional Review Board of Taichung Veterans General Hospital in Taiwan.

## Supplementary Materials

The following are available online at https://www.mdpi.com/article/10.3390/jcm10112323/s1, Table S1: ROC curve analysis of parameters with significant differences between the survivors and nonsurvivors.
