# Supplementary material for: Prognostic Factors to Predict ICU Mortality in Patients with Severe ARDS Who Received Early and Prolonged Prone Positioning Therapy
Source: J Clin Med. 2021 May 26;10(11):2323. doi: 10.3390/jcm10112323 (PMC8198972; doi:10.3390/jcm10112323)
Supplement: Supplementary file 1 [file jcm-10-02323-s001.zip › jcm-1226632-supplementary.pdf]

Supplement Table 1. ROC curve analysis of parameters with significant differences between the survivors and nonsurvivors

Characteristics	AUC	*p* value	Cut-off point	Sensitivity	Specificity	Accuracy	PPV	NPV
Prone score	0.816	<0.001**	≥3	75.0	82.7	78.5	84.2	72.9
Age	0.668	0.001**	≥53	89.1	44.2	69.0	66.3	76.7
APACHE II	0.623	0.018 *	≥33	48.4	76.9	61.2	72.1	54.8

APACHE II: Acute Physiology and Chronic Health Evaluation I; AUC, area under the curve; NPV, negative predictive value; PPV, positive predictive value; ROC, receiver operating characteristic. *p < 0.05, **p < 0.01.
